# Improving Sensitivity and Resolution of Dendrimer Identification in MALDI-TOF Mass Spectrometry Using Varied Matrix Combinations

**DOI:** 10.3390/polym17020219

**Published:** 2025-01-16

**Authors:** Claudia Sanhueza, Nathalia Baptista Dias, Daniela Vergara, Lisette Silva, Emigdio Chávez-Ángel, Alejandro Castro-Alvarez

**Affiliations:** 1 Center for Resilience, Adaptation and Mitigation (CReAM), Faculty of Sciences, Universidad Mayor, Temuco 4780000, Chile; 2 Escuela de Ingeniería, Facultad de Ciencias, Ingeniería y Tecnología, Universidad Mayor, Temuco 4780000, Chile; 3 Scientific and Technological Bioresource Nucleus (BIOREN-UFRO), Universidad de La Frontera, Temuco 4811230, Chile; nathalia.dias@ufrontera.cl; 4 Centro de Excelencia en Medicina Traslacional (CEMT), Facultad de Medicina, Universidad de La Frontera, Temuco 4811230, Chile; daniela.vergara@ufrontera.cl; 5 Carrera de Química y Farmacia, Facultad de Medicina, Universidad de La Frontera, Temuco 4811230, Chile; 6 Catalan Institute of Nanoscience and Nanotechnology, CSIC and BIST, Campus UAB, 08193 Bellaterra, Barcelona, Spain; 7 Departamento de Ciencias Preclínicas, Facultad de Medicina, Universidad de La Frontera, Temuco 4811230, Chile

**Keywords:** dendrimers, MALDI, poly-amidoamine, polymer analysis

## Abstract

Matrix-assisted laser desorption/ionization time-of-flight mass spectrometry (MALDI-TOF MS) is a well-known technique for polymer analysis, particularly for determining the molecular weight and structural details of dendrimers. In this study, we evaluated the performance of various matrices, such as 2′,4′,6′-trihydroxyacetophenone (THAP), α-cyano-4-hydroxycinnamic acid (HCCA), and sinapinic acid (SA), and their combinations, on the sensitivity and resolution of poly(amidoamine) (PAMAM) dendrimers of different generations (G3.0, G4.0, and G5.0). Our results demonstrated that the combination of HCCA-THAP significantly enhanced spectral resolution and peak intensity compared to individual matrices, particularly for higher-generation dendrimers. This improvement is attributed to the better ionization efficiency achieved with the combined matrices. These findings provide critical insights into optimizing MALDI-TOF MS for the accurate characterization of complex polymers, with potential applications in drug delivery and nanotechnology.

## 1. Introduction

Matrix-assisted laser desorption/ionization time-of-flight mass spectrometry (MALDI-TOF/MS) has emerged as a versatile, robust, and cost-effective analytical technology for molecular mass determination, offering rapid analysis with high sensitivity and minimal sample consumption [1]. This technique is widely used/employed in protein and polymer structure analysis research, encompassing parameters such as the number of repeating polymer units, molecular weight distribution, terminal structure, and other essential information.

Various polymers have been successfully analyzed using MALDI-TOF/MS, including poly(N-vinylpyrrolidone) (PVP) [2], which has been characterized for molecular weight distribution and terminal functionalization, and polyflavonoid tannins [3], where MALDI-TOF elucidated polymerization patterns and specific monomeric units.

Compared to traditional structural analysis techniques, such as nuclear magnetic resonance (NMR) and X-ray diffraction (XRD), MALDI-TOF/MS offers faster sample preparation and requires significantly lower quantities of material [4]. While MALDI-TOF/MS excels in providing rapid insights into molecular weights and oligomeric structures, NMR and XRD remain indispensable for elucidating detailed molecular structures and crystallinity.

These techniques are highly complementary; MALDI-TOF/MS efficiently provides molecular weight distribution and terminal structure data, which can be corroborated with NMR for functional group analysis or XRD for crystalline phase identification. By leveraging the unique strengths of each method, a comprehensive understanding of synthetic and natural polymers can be achieved.

**Figure 1:**
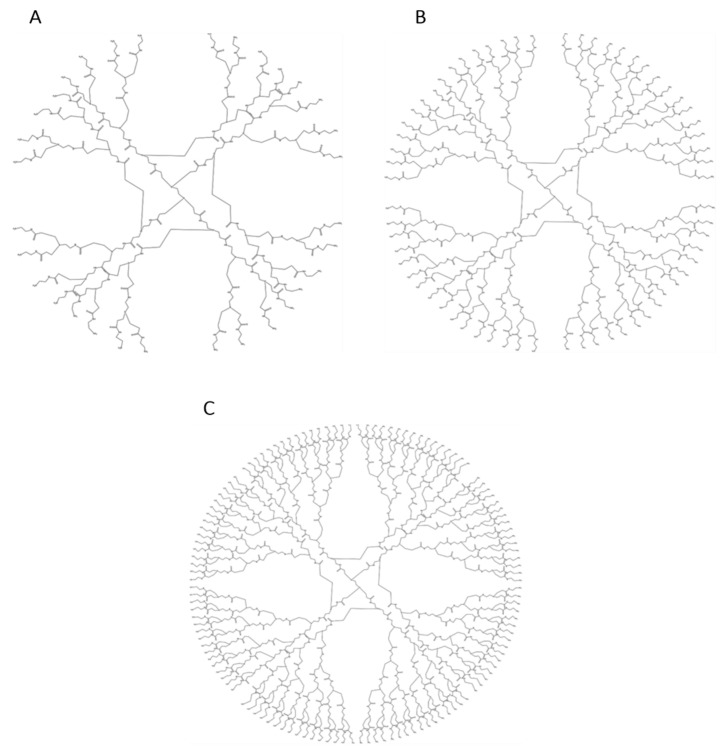
Chemical structure in 2D of PAMAM dendrimers of (**A**) third (G3.0), (**B**) fourth (G4.0), and (**C**) fifth (G5.0) generations, all with an ethylenediamine (EDA) core. The terminal groups on each dendrimer increase with each generation, allowing for versatile functionalization potential, which is useful in drug delivery and nanomedicine applications.

Dendrimers are three-dimensional (3D) branched polymers with tree-like architectures (Figure 1). Their structures consist of repetitive branched units arising from a central core, creating successive branches in concentric layers. These unique structures impart distinctive properties, including nanosized frameworks, high charge density, and high solubility. These characteristics make them an ideal platform for the encapsulation of a wide range of bioactive compounds with diverse chemical properties. Their complex structures form a family apart from linear or conventionally branched molecules [5]. Dendrimers possess several functional end groups responsible for high solubility and high reactivity and empty internal cavities, making them suitable carriers for drugs and nucleic acids [6]. Their high water solubility, particularly among cationic dendrimers, enables them to be effective nanocarriers for biological applications [7].

In particular, poly(amidoamine) (PAMAM) dendrimers have been the most extensively studied due to their well-defined structure, biocompatibility, and ease of functionalization. The surface functional groups of PAMAM dendrimers can be modified to enhance their interaction with specific biological targets, improving their efficacy as drug delivery systems [8,9]. These dendrimers have been widely used for the targeted delivery of drugs, genes, and other therapeutic agents due to their ability to encapsulate or bind molecules within their internal cavities or on their surfaces [10]. For instance, their cationic nature facilitates the formation of complexes with negatively charged nucleic acids, aiding in gene delivery by protecting the delicate structure of nucleic acids from degradation and promoting cellular uptake [11]. The structural variations across different generations of PAMAM dendrimers are illustrated in Figure 1.

However, the determination of polymer molecular weight via MALDI-TOF presents challenges. Natural and synthetic polymers show an intricate distribution of chain lengths that leads to their intrinsic polydisperse nature, which may result in an underestimation of the high mass tail [12]. The complex 3D-branched structure in dendrimers is fragile and prone to decomposition during ionization, affecting measurement precision. Additionally, dendrimers may have multiple functional groups with positive or negative charges, resulting in multiple ionic species that complicate mass spectra interpretation.

The choice of matrix for ionization in MALDI-TOF significantly influences the method efficiency and reproducibility, with some dendrimers showing greater sensitivity to matrix conditions than others. The matrix plays a crucial role in assisting the ionization process. Three key functions of the matrix have been suggested: incorporation of the analyte within the matrix crystals, a collective absorption and ablation event upon laser irradiation, and an active role of the matrix in promoting ionization of the analyte. Ideally, the matrix should possess specific properties to ensure efficient ionization and high-quality spectra: it should have a high electronic absorption at the laser wavelength, good vacuum stability, low vapor pressure, high solubility in solvents compatible with the analyte, and good miscibility with the analyte in the solid state [13]. These characteristics help optimize analyte incorporation, ionization efficiency, and spectrum reproducibility, which is particularly important when analyzing large and complex molecules like dendrimers. Several matrices have been identified for practical dendrimer analysis: THAP (2′,4′,6′-trihydroxyacetophenone) is commonly used and has shown favorable results for intermediate PAMAM generations [14,15,16]. Other matrices, such as 2,5-dihydroxybenzoic acid (DHB) and DCTB (2-[(2E)-3-(4-tert-butylphenyl)-2-methylprop-2-enylidene] malononitrile), have also been employed in studies to improve ionization for specific dendrimer types or higher generations [6,7,17]. However, further research is needed to optimize matrix selection, particularly as dendrimer generations increase in size and complexity.

In this study, we aimed to evaluate the performance of different matrices for enhancing the sensitivity and accuracy of molecular mass determination of PAMAM dendrimers across different generations (G3.0, G4.0, and G5.0) using MALDI-TOF. This investigation sought to refine the analysis protocol for dendrimers, addressing current challenges in high-mass polymer detection and providing a reliable framework for their characterization.

## 2. Materials and Methods

PAMAM dendrimers of G3.0, G4.0, and G5.0 in methanol were purchased from Dendritech Inc. (Midland, MI, USA) and were used without further purification. The average molecular weights of G3.0, G4.0, and G5.0 were 6908 g/mol, 14,214 g/mol, and 28,824 g/mol, respectively. For MALDI-TOF analysis, 2,4,6-trihydroxyacetophenone (THAP; CAS Number 249278-28-2), α-cyano-4-hydroxycinnamic acid (HCCA; CAS Number 28166-41-8), Sinapinic acid (SA; CAS Number 530-59-6), acetonitrile (CAS Number 75-05-8), trifluoroacetic acid (TFA; CAS Number 76-05-1), and ultrapure water, all of MALDI-TOF grade, were purchased from Sigma-Aldrich (Darmstadt, Germany).

Each matrix (HCCA, THAP, and SA) was individually prepared at a saturated concentration of 20 mg/mL in a solution of 50% (*v*/*v*) acetonitrile and 50% (*v*/*v*) TFA-acidified water containing 0.1% (*v*/*v*) TFA. Conventional acidic matrices often face challenges in providing an effective ionization strategy for polymers. Therefore, selecting a suitable cationization agent is crucial [4]. In this case, the cationization agent selected was TFA to promote protonation and maximize ionization efficiency. The matrices were then combined at a final concentration of 10 mg/mL each, in a 1:1 ratio, to assess the effects of different matrix combinations on dendrimer ionization. The specific combinations tested were HCCA-SA, THAP-SA, and HCCA-THAP.

For sample preparation, PAMAM dendrimers were initially evaluated at two concentrations: 1 and 10 mg/mL in a solution of 50% (*v*/*v*) acetonitrile and 50% (*v*/*v*) TFA-acidified water containing 0.1% (*v*/*v*) TFA. However, the 1 mg/mL concentration was too low, resulting in no detectable peaks in the spectra. Higher concentrations were not tested, as the drying process became challenging at higher matrix concentrations, leading to non-uniform co-crystallization of the polymer on the MALDI plate. Consequently, all analyses were conducted using 10 mg/mL of dendrimers to ensure sufficient signal intensity.

A 1 μL aliquot of each dendrimer solution was carefully applied to a polished steel MALDI-TOF sample plate (Bruker Daltonics, Bremen, Germany) and allowed to dry at room temperature. After drying, 1 μL of the prepared matrix solution was applied directly onto each dendrimer spot. Samples were left to air-dry again, forming co-crystallized spots suitable for MALDI-TOF analysis. Mass spectra were acquired in linear and positive ion modes using a MALDI-TOF Autoflex Speed instrument (Bruker Daltonics, Bremen, Germany) equipped with a 355 nm Smartbeam laser source. The laser intensity was set to 70% after a routine laser energy optimization, which was performed to select the lowest energy that could effectively ionize the sample without causing its destruction. This approach ensured sufficient ionization while preserving the integrity of the sample.

The use of characterized commercial polymers in our study served to standardize the MALDI-TOF protocol by minimizing noise introduced by impurities and ensuring the reliability of the method. This approach allowed us to validate the protocol’s ability to accurately determine the molecular weight of PAMAM dendrimers, which is critical for subsequent analyses.

All samples were analyzed in triplicate. Each spectrum was collected as an average of 1200 laser shots, with enough energy to generate clear spectra, avoiding the saturation of the sensor. The mass-to-charge ratio (*m*/*z*) range was 800 to 100,000 Da, examined across multiple experiments and mass spectrometry acquisitions. The standard used contains insulin, ubiquitin, cytochrome C, and myoglobin and was specifically designed to calibrate method parameter files for MALDI-TOF mass spectrometers. It ensures accurate performance within a low mass range of 4000 to 20,000 Da. This standard was selected due to its compatibility with the mass range of the evaluated polymers, which fall between 7000 and 28,000 Da.

## 3. Results

**Figure 2:**
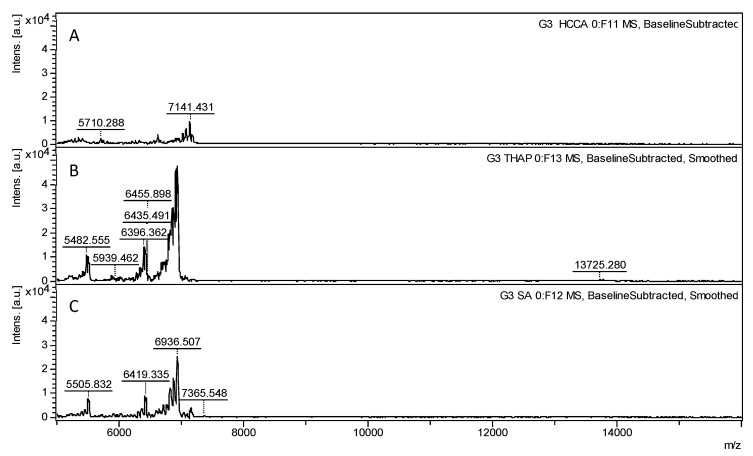
MALDI-TOF spectra of G3.0 PAMAM dendrimers treated with different matrices. The spectra displayed a mass-to-charge (*m*/*z*) range from 5000 to 7500 *m*/*z*: (**A**) HCCA, (**B**) THAP, and (**C**) SA. Each spectrum demonstrates how distinct matrices affect ionization and fragmentation of the dendrimer structure, underlining the importance of matrix selection for optimizing the mass spectral analysis of PAMAM dendrimers.

Three matrices (THAP, HCCA, and SA) were evaluated to investigate their impact on the detection and resolution of PAMAM dendrimer spectra in MALDI-TOF. Figure 2 shows the MALDI-TOF spectra for PAMAM G3.0 with an ethylenediamine core, analyzed for each HCCA, THAP, and SA matrix. Each spectrum shows multiple peaks corresponding to various molecular weights, with the peak at 6936 *m/z* consistently exhibiting the highest intensity across all spectra.

The calculated molecular weight of PAMAM G3.0, approximately 6908 g/mol, closely aligns with the observed values. While MALDI-TOF is known for its precision in determining molecular weights, dendrimers, such as PAMAM, tend to show a distribution of molecular weights rather than a singular value. This distribution may result from synthesis-related defects, such as missing branches or intramolecular cyclizations, where two branches are connected by a secondary amine [18]. Additionally, thermal reactions during the matrix’s ionization process could further contribute to these variations [19], despite the otherwise clean synthesis process being efficient and yielding the desired product.

Figure 2 also reveals a general trend of low peak intensities in the spectra, with PAMAM G3.0 reaching 28,381 a.u. when HCCA was used as the matrix. The SA matrix produced a slightly lower peak intensity of 25,538 a.u. These relatively low signal intensities reflect the typical challenges encountered in the MALDI-TOF analysis of dendrimers, where the desorption/ionization of such large macromolecules is often complex. However, a notable improvement in signal intensity was observed with the THAP matrix, yielding a significantly higher peak intensity of 48,000 a.u. for G3.0. All the peaks with their corresponding intensity values are provided in the Supplementary Materials, in Table S1 for HCCA, Table S2 for THAP, and Table S3 for SA.

**Figure 3:**
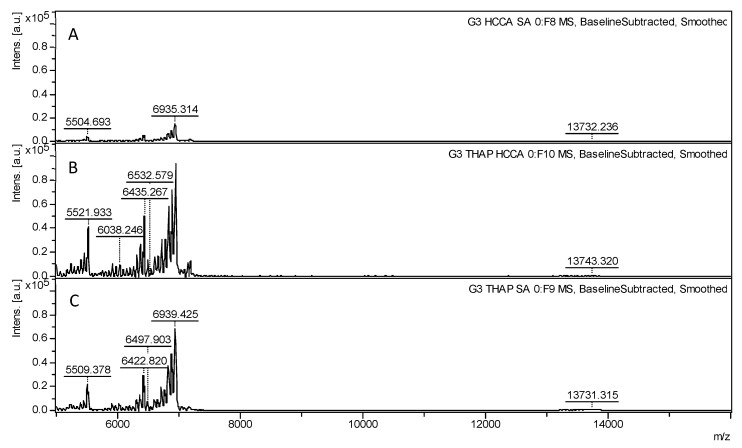
MALDI-TOF spectra of G3.0 PAMAM dendrimers treated with different matrix combinations, covering a mass-to-charge (*m*/*z*) range from 5000 to 7500 *m*/*z*. (**A**) HCCA-SA combination results in a spectrum with clear peak distribution but comparatively lower intensity. (**B**) HCCA- THAP shows a significant increase in peak intensity, highlighting enhanced ionization efficiency with this blend. (**C**) THAP-SA.

Various matrix combinations were tested to address this problem and further enhance the spectral intensity for G3.0, including HCCA-SA, HCCA-THAP, and THAP-SA, as shown in Figure 3. The combination of THAP and HCCA resulted in a significant increase in intensity, reaching 94,000 a.u., which is a significant improvement compared to the 14,582 a.u. observed for the HCCA-SA combination. The intensity of the HCCA-THAP combination surpassed the values obtained when either matrix was used individually. The THAP-SA combination also produced a maximum intensity of 69,482 a.u. at the 6935 *m*/*z* peak, demonstrating that matrix combinations involving THAP can enhance peak intensity. In contrast, combinations that excluded THAP, such as HCCA-SA, led to a large reduction in sensitivity, as evidenced by the low intensity of 14,582 a.u. at the 6935 *m*/*z* peak for the G3.0 dendrimer. All the peaks with their corresponding intensity values are provided in the Supplementary Materials, in Table S4 for HCCA-SA, Table S5 for HCCA-THAP, and Table S6 for THAP-SA.

**Figure 4:**
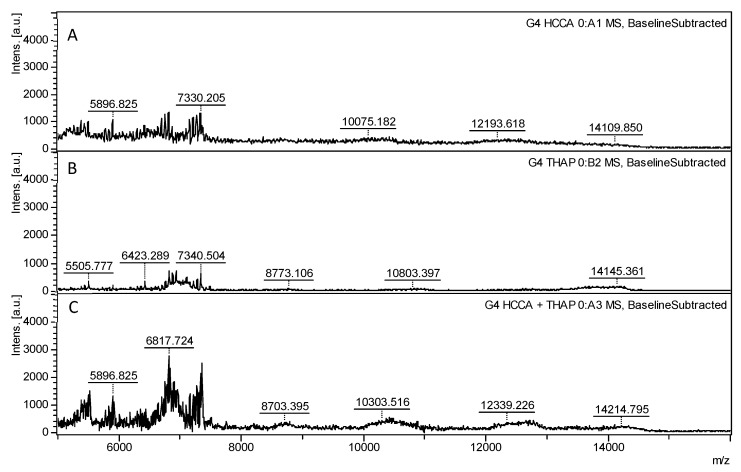
MALDI-TOF spectra of 4.0 PAMAM dendrimers analyzed with the most effective individual matrices and the optimal matrix combination identified from G3.0 analysis, displayed over a mass-to-charge (*m*/*z*) range of 5000 to 20,000. The spectra reveal how each matrix setup influences ionization and the spectral quality of G4.0 PAMAM dendrimers. (**A**) THAP produces a well-defined spectrum with distinct peak clarity, providing baseline ionization efficiency for comparison. (**B**) HCCA yields a comparable spectral profile with moderate peak intensities, supporting a reliable analysis of the dendrimer structure. (**C**) HCCA-THAP, the combination that demonstrated optimal peak intensity in G3.0 analysis, results in a markedly enhanced spectrum with high peak intensities and resolution, confirming its effectiveness for detailed structural characterization.

**Figure 5:**
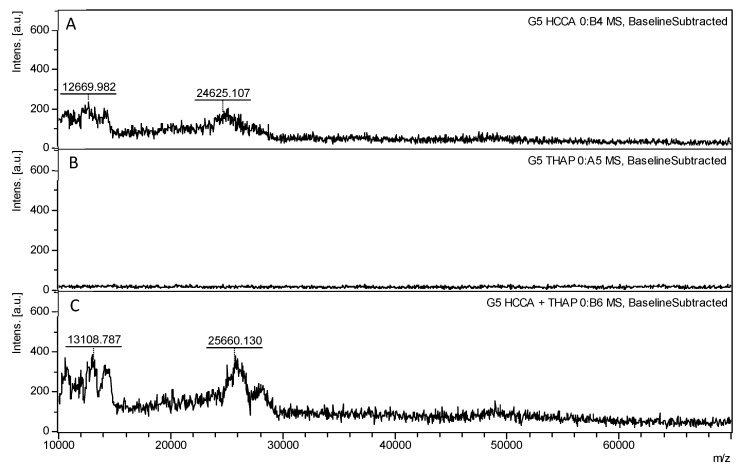
MALDI-TOF spectra of G5.0 PAMAM dendrimers were analyzed using the most effective individual matrices and the optimal matrix combination identified from G3.0 analysis, covering a mass-to-charge (*m*/*z*) range of 10,000 to 80,000. The spectra illustrate the impact of matrix choice on ionization and spectral quality for G5.0 dendrimers, whose molecular weight is approximately double that of G4.0 PAMAM dendrimers and four times that of third-generation (G3.0) dendrimers. (**A**) THAP. (**B**) HCCA. (**C**) HCCA-THAP combination, identified as optimal for G3.0, achieves significantly higher peak intensity and resolution, enabling a detailed structural characterization of G5.0 PAMAM dendrimers across this higher *m*/*z* range. This enhanced spectrum underscores the effectiveness of HCCA-THAP for high-generation PAMAM dendrimer analysis, emphasizing its role in improving data quality for larger molecular structures.

Based on these results, this study identified the HCCA-THAP matrix combination as the most effective for enhancing spectral intensity and resolution. Consequently, this combination was selected to analyze higher-generation PAMAM dendrimers, specifically G4.0 (Figure 4) and G5.0 (Figure 5).

For PAMAM G4.0, the theoretical molecular weight is approximately 14,200 g/mol, with corresponding masses detected using both the THAP matrix and the HCCA-THAP combination, yielding intensities of 174 a.u. and 274 a.u., respectively (Figure 4). When HCCA was used as the sole matrix, a broad peak centered at approximately 12,000 *m*/*z* with an intensity of 372 a.u. was observed. In contrast to the behavior observed for G3.0, the intensity of this peak was larger for G4.0. The linewidth of the peak is closely related to its theoretical molecular weight (see Tables S7–S9).

The combined HCCA-THAP matrix produced peaks that were both narrower and well-defined. The characteristic peak of G4.0 was observed at 14,108 *m*/*z*, which is much closer to the theoretical mass of the polymer, highlighting an improvement in resolution and sensitivity with the combined matrix. These findings suggest that incorporating THAP and HCCA together yields more defined and accurate peaks for PAMAM dendrimers of both the third and fourth generations. However, a decrease in sensitivity was observed as the molecular weight increased, evidenced by the reduction in peak intensities. This decrease reflects the challenges of ionizing larger polymers, where MALDI-TOF struggles to maintain efficiency.

No peak was observed with THAP alone for PAMAM G5.0 (Figure 5), further demonstrating that higher molecular weights make ionization more difficult. The theoretical molecular weight of PAMAM G5.0 is 28,824 g/mol, and closer masses were observed using HCCA, with a broad peak between 25,025 and 28,736 *m*/*z*, reaching intensities of 219 a.u. and 124 a.u., respectively. When HCCA and THAP were combined, as seen in G4.0, the spectrum for G5.0 was more defined and sensitive, showing two narrow peaks at 25,071 and 27,948 *m*/*z*, with intensities of 388 a.u. and 242 a.u., respectively. These results strongly suggest that the combination of both matrices (HCCA-THAP) significantly increases the intensity of the peaks, indicating enhanced ionization efficiency and sensitivity for higher-molecular-weight PAMAM dendrimers (see Tables S10 and S11).

## 4. Discussion

This study explored the impact of different matrix combinations on the sensitivity and resolution of MALDI-TOF for the analysis of PAMAM dendrimers. As a small molecule, the matrix is critical in absorbing laser energy and facilitating proton transfer during the MALDI process. The solubility of the matrix, relative to the hydrophilicity or hydrophobicity of the polymer, significantly influences the uniformity of the mixture. When the solubility properties of the matrix closely match those of the polymer, it enhances the uniformity of the solution and promotes consistent co-crystallization on the MALDI target. This uniformity is essential for ensuring reliable ionization, reproducible spectra, and accurate molecular weight determination during MALDI-TOF analysis [4]. By comparing the performance of individual matrices and combinations, we aimed to identify an optimal setup for improved ionization efficiency and data quality across three different dendrimer generations. Our results significantly improved signal intensity and peak resolution when using the HCCA-THAP matrix combination, especially for G3.0 dendrimers. These findings contribute to optimizing MALDI-TOF analysis of dendrimers and other complex polymers, making molecular weight determination more accurate.

**Figure 6:**
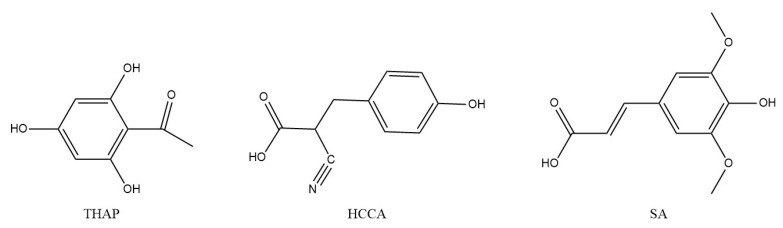
Chemical structure of the matrix used in this study.

The chemical structures of the matrices differed significantly, with the benzene group being the only common feature. However, their substitutions varied considerably, resulting in distinct chemical properties, including pKa values, which influenced the solubility and affinity for the functional groups of the dendrimers and thus the co-crystallization process on the plate. Generally, polymers with high proton affinities can be protonated by matrices containing carboxylic acid groups [13,20]. In this study, THAP was the only matrix containing no carboxylic acid groups. Its structure includes three hydroxyl groups and one ketone group, which facilitates polymer ionization, showing good performance with G3.0 dendrimers. However, as molecular weight increases, its efficiency in promoting ionization decreases. Conversely, HCCA contains a carboxyl group and a highly electronegative cyano group. The cyano group increases the hydroxyl group’s acidity and enhances the carboxylic group’s acidity through an inductive effect, likely contributing significantly to polymer ionization (Figure 6). HCCA performed better with G4.0 and G5.0 dendrimers than with THAP alone, and the combination of THAP and HCCA demonstrated a synergistic effect on dendrimer ionization. Finally, SA contains two ether groups, one hydroxyl group, and one carboxyl group. The carboxyl group in SA is less acidic than that in HCCA, likely because of the electron-donating effects of the ether groups in its structure.

Furthermore, despite the high efficiency of HCCA and THAP, the increasing molecular weight of dendrimer generation led to decreased ionization efficiency and reduced vaporization of the molecules.

Several studies have addressed the complexity of PAMAM dendrimer analysis using MALDI-TOF. Like most polymers, dendrimers exhibit a broad distribution of molecular weights due to synthesis imperfections and fragmentation during ionization. Subbi et al. [21] identified multiple fragmentation pathways influenced by matrix choice and laser pulse energy, which explains the sensitivity variance we observed between different matrices [21]. In our study, the HCCA-THAP combination showed superior performance in enhancing signal intensity, which aligns with previous reports, including So et al.’s [12], where matrix selection was critical in determining fragmentation efficiency in PAMAM dendrimers.

Our results showed that THAP, either alone or in combination with HCCA, enhanced the peak intensities, consistent with the findings of Zhou et al. [14], who demonstrated that THAP, along with other matrices, provided accurate molecular weight determination for PAMAM dendrimers encapsulating metal ions. The increased signal intensity observed with the HCCA-THAP combination might be attributed to enhanced protonation, which facilitates the desorption and ionization of larger dendritic structures. This aligns with previous studies by Redding et al. [18] and Wang et al. [22], who emphasized the matrix influence in improving ionization efficiency in dendrimer studies.

A significant limitation of this study was the decreased sensitivity of higher-generation dendrimers, a trend also highlighted by Hernández-Ramirez et al. [23] in their analysis of PAMAM dendrimers. Higher-generation dendrimers, such as G4.0 and G5.0, tend to have more complex structures, making ionization more difficult. This issue was also noted by Leriche et al. [17], who showed that dendrimers with functionalized end groups exhibited complex fragmentation patterns [17]. The fragmentation patterns we observed were consistent with the findings of Myung et al. [24], who reported that the branching architecture and surface functionalization of dendrimers play a significant role in their ionization and fragmentation behaviors [24].

Moreover, our study reaffirms the importance of matrix combinations for overcoming the limitations of MALDI-TOF when analyzing larger macromolecules. The combination of THAP and HCCA matrices produced the highest intensities and provided clearer spectra for dendrimer analysis, making it possible to overcome some of the inherent challenges associated with dendrimer polydispersity and fragmentation. Wang et al. previously demonstrated the utility of MALDI-TOF in visualizing dendrimer structures, highlighting its potential for advanced biomedical applications [22]. Our findings support these observations, suggesting that optimized matrix selection could enhance MALDI-TOF’s utility for biomedical applications that rely on accurate dendrimer characterization, such as drug delivery and gene therapy.

Finally, our findings also contribute to this field of research, suggesting that dendrimers, with their unique branched structures, can be optimized for various technological applications. For instance, dendrimers have been shown to encapsulate and stabilize metal ions, a property that could be further explored in drug delivery systems or catalysis, as noted by Zhou et al. [14]. The advances in MALDI-TOF techniques, particularly matrix selection, will facilitate the further development of dendrimer-based technologies, enhancing their applicability in fields ranging from nanomedicine to environmental remediation [14,23].

## 5. Conclusions

In this study, we have demonstrated the critical influence of matrix selection on the sensitivity and resolution of PAMAM dendrimer characterization using MALDI-TOF. The combination of THAP and HCCA matrices significantly enhanced the signal intensities for G3.0 PAMAM dendrimers, yielding superior spectral resolution and peak intensities compared to single-matrix applications. Our findings confirm that the HCCA-THAP combination, each at a concentration of 10 mg/mL, is the most effective matrix system for dendrimer analysis across the three generations evaluated. This combination not only improved the precision of molecular weight determination but also showed a marked improvement in resolution for higher-generation dendrimers (such as G4.0 and G5.0, tested in this study), which are generally more challenging to analyze due to their structural complexity and increased polydispersity.

We propose several enhancements for future studies to address the acknowledged challenges of high-mass dendrimer analysis. Investigating new or tailored matrix combinations designed to improve ionization efficiency for high-mass polymers like PAMAM could improve performance, particularly with matrices offering enhanced co-crystallization properties. Advanced sample preparation techniques, such as layered matrix deposition and optimized co-crystallization conditions, could ensure uniform distribution and reduce noise from matrix inhomogeneities. Adjustments to instrument settings, including laser energy, ion extraction times, or delayed extraction modes, may optimize the ionization of high-mass dendrimers without causing fragmentation.

This work provides valuable insights for optimizing MALDI-TOF conditions in polymer science, underscoring the importance of tailored matrix selection for complex macromolecules. MALDI-TOF MS matrix optimization advancements are expected to support more accurate and efficient dendrimer analyses, opening pathways for their expanded use in applications such as drug delivery, nanotechnology, and biomedicine, where precise control over molecular architecture is crucial.

## Data Availability

Data are contained within the article and Supplementary Materials.

## Supplementary Materials

The following supporting information can be downloaded at https://www.mdpi.com/article/10.3390/polym17020219/s1. Table S1: Supplementary Data from MALDI-TOF Analysis of PAMAM G3.0 Using HCCA Matrix. Table S2: Supplementary Data from MALDI-TOF Analysis of PAMAM G3.0 Using THAP Matrix. Table S3: Supplementary Data from MALDI-TOF Analysis of PAMAM G3.0 Using SA. Table S4: Supplementary Data from MALDI-TOF Analysis of PAMAM G3.0 Using a Combination of HCCA-SA. Table S5: Supplementary Data from MALDI-TOF Analysis of PAMAM G3.0 Using a Combination of THAP-HCCA. Table S6: Supplementary Data from MALDI-TOF Analysis of PAMAM G3.0 Using a Combination of THAP-SA. Table S7: Supplementary Data from MALDI-TOF Analysis of PAMAM G4.0 Using HCCA as Matrix. Table S8: Supplementary Data from MALDI-TOF Analysis of PAMAM G4.0 Using THAP as Matrix. Table S9: Supplementary Data from MALDI-TOF Analysis of PAMAM G4.0 Using a Combination of HCCA-THAP. Table S10: Supplementary Data from MALDI-TOF Analysis of PAMAM G5.0 Using HCCA as Matrix. Table S11: Supplementary Data from MALDI-TOF Analysis of PAMAM G5.0 Using a Combination of HCCA-THAP.
