# Supplementary material for: Improving Sensitivity and Resolution of Dendrimer Identification in MALDI-TOF Mass Spectrometry Using Varied Matrix Combinations
Source: Polymers (Basel). 2025 Jan 16;17(2):219. doi: 10.3390/polym17020219 (PMC11769133; doi:10.3390/polym17020219)
Supplement: Supplementary file 1 [file polymers-17-00219-s001.zip › polymers-3337407-supplementary.pdf]

Table S1. Supplementary Data from MALDI-TOF Analysis of PAMAM G3.0 Using HCCA Matrix.

m/z	time	Intens.	SN	Res.	Area	Rel. Intens.	FWHM
5009.471	47831.84	1377.500	8.366	394.360	15869.731	0.049	12.703
5026.383	47911.27	1210.500	7.352	477.262	9637.209	0.043	10.532
5040.063	47975.42	848.500	5.153	902.298	4460.350	0.030	5.586
5070.419	48117.45	889.500	5.402	1092.203	4195.716	0.031	4.642
5081.733	48170.28	984.500	5.979	537.566	4008.441	0.035	9.453
5126.328	48377.95	1383.500	8.692	880.493	10743.787	0.049	5.822
5131.220	48400.67	1544.500	9.704	2115.758	8386.119	0.055	2.425
5142.612	48453.55	1289.500	8.102	738.589	12068.045	0.046	6.963
5155.943	48515.35	1238.500	7.781	1207.182	6180.487	0.044	4.271
5183.788	48644.19	1782.500	11.199	661.257	16322.650	0.063	7.839
5194.459	48693.48	2133.500	13.405	2959.257	10319.544	0.075	1.755
5199.229	48715.49	2125.500	13.354	1656.803	12702.417	0.075	3.138
5213.041	48779.17	1524.500	9.863	1247.388	9873.418	0.054	4.179
5222.577	48823.09	1671.500	10.814	799.377	13813.840	0.059	6.533
5243.504	48919.33	3118.500	20.175	645.145	37337.222	0.110	8.128
5255.901	48976.26	3343.500	21.631	910.656	32764.381	0.118	5.772
5269.665	49039.38	2836.500	18.351	782.258	26093.476	0.100	6.736
5282.190	49096.75	1279.500	8.278	952.929	8015.003	0.045	5.543
5291.629	49139.93	973.500	6.298	2003.390	3058.530	0.034	2.641
5298.504	49171.36	1219.500	7.890	835.464	8709.164	0.043	6.342
5306.320	49207.07	926.500	6.165	3379.007	2665.447	0.033	1.570
5313.445	49239.60	1164.500	7.748	828.065	8277.257	0.041	6.417
5327.453	49303.49	1217.500	8.101	1358.698	5413.393	0.043	3.921
5370.968	49501.43	1090.000	7.253	1061.111	6339.110	0.038	5.062
5412.891	49691.37	1881.000	12.866	652.091	16632.974	0.066	8.301
5468.434	49941.88	1512.500	10.345	464.614	11322.684	0.053	11.770
5501.422	50090.07	946.500	6.474	704.905	3954.134	0.033	7.804
5527.775	50208.13	6065.500	42.551	563.105	66501.700	0.214	9.817
5543.701	50279.34	3093.000	21.698	684.686	36010.121	0.109	8.097
5657.732	50786.26	1056.250	7.588	3395.257	4199.777	0.037	1.666
5728.385	51097.78	828.000	6.101	971.229	4472.985	0.029	5.898
5813.474	51470.42	1271.000	9.365	654.206	10815.095	0.045	8.886
5826.120	51525.56	1142.000	8.415	668.267	10217.320	0.040	8.718
5840.379	51587.67	890.250	6.711	963.861	5330.323	0.031	6.059
5868.099	51708.20	1064.000	8.021	1774.581	3421.530	0.038	3.307
5927.064	51963.64	2331.500	17.576	632.200	33301.197	0.082	9.375
5942.311	52029.48	2365.500	18.231	1207.284	25080.344	0.084	4.922
5955.672	52087.11	1268.500	9.776	948.382	8181.725	0.045	6.280
5961.240	52111.11	877.500	6.763	1950.662	3362.432	0.031	3.056
5985.697	52216.38	2252.500	17.360	824.000	25038.974	0.080	7.264
5999.678	52276.46	1563.500	12.050	1017.936	13588.082	0.055	5.894
6013.320	52335.02	944.500	7.279	851.498	8363.272	0.033	7.062

6028.058	52398.21	1352.500	10.424	1181.155	8513.746	0.048	5.104
6045.187	52471.55	3664.500	28.243	687.104	49674.884	0.129	8.798
6060.385	52536.54	2596.500	20.401	1100.247	26104.868	0.092	5.508
6068.981	52573.26	2285.500	17.958	874.406	20956.410	0.081	6.941
6083.290	52634.33	1436.500	11.287	2248.420	8467.280	0.051	2.706
6100.593	52708.08	2684.500	21.093	860.555	28461.869	0.095	7.089
6113.749	52764.08	2543.500	19.985	1652.376	24552.558	0.090	3.700
6127.075	52820.75	2292.500	18.013	1109.321	22565.632	0.081	5.523
6142.929	52888.09	2101.500	16.512	1446.376	19555.956	0.074	4.247
6159.724	52959.33	3469.500	27.708	658.352	45489.404	0.123	9.356
6171.696	53010.05	3437.500	27.452	831.287	38090.301	0.121	7.424
6185.098	53066.77	3093.500	24.705	622.910	41375.786	0.109	9.929
6198.456	53123.25	1629.500	13.013	831.087	11982.869	0.058	7.458
6214.164	53189.58	2388.500	19.075	841.891	25293.311	0.084	7.381
6229.766	53255.38	1740.500	13.900	906.568	17358.459	0.061	6.872
6242.667	53309.73	1537.500	12.279	1198.599	14351.029	0.054	5.208
6259.994	53382.63	1610.500	12.862	980.723	11178.861	0.057	6.383
6270.258	53425.77	1796.500	14.558	764.251	20632.504	0.063	8.204
6286.833	53495.36	1723.500	13.966	1043.793	18200.415	0.061	6.023
6329.070	53672.27	3025.500	24.517	658.002	30755.324	0.107	9.619
6342.272	53727.45	1552.500	12.581	750.652	14087.885	0.055	8.449
6384.468	53903.42	4053.500	33.374	679.968	41356.847	0.143	9.389
6398.911	53963.52	2009.500	16.545	810.353	19751.838	0.071	7.896
6416.725	54037.55	1436.500	11.827	825.207	14021.698	0.051	7.776
6443.679	54149.37	9492.500	78.155	737.183	106980.351	0.335	8.741
6459.255	54213.88	4524.500	37.252	930.759	38615.899	0.160	6.940
6502.683	54393.34	3138.000	26.283	675.464	35420.460	0.111	9.627
6519.330	54461.97	1851.250	15.506	623.425	16742.647	0.065	10.457
6557.836	54620.38	1565.000	13.108	776.899	10449.463	0.055	8.441
6572.349	54679.97	913.500	7.651	843.100	5467.014	0.032	7.795
6617.719	54865.82	4746.125	40.385	642.535	59441.877	0.168	10.299
6630.785	54919.22	3491.125	29.706	815.975	45438.251	0.123	8.126
6644.426	54974.92	2223.125	18.917	774.978	18359.042	0.078	8.574
6672.570	55089.66	1885.125	16.041	960.728	12528.101	0.067	6.945
6687.634	55150.97	1106.125	9.412	1342.141	2792.248	0.039	4.983
6702.987	55213.39	962.125	8.187	914.346	5069.845	0.034	7.331
6732.522	55333.26	6005.125	51.755	661.643	85891.338	0.212	10.175
6746.478	55389.81	3706.125	31.941	1175.870	38170.664	0.131	5.737
6786.468	55551.53	3857.125	33.242	672.254	31161.864	0.136	10.095
6802.515	55616.29	1796.125	15.480	1282.240	9869.762	0.063	5.305
6822.346	55696.22	1002.125	8.637	596.874	7807.784	0.035	11.430
6823.686	55701.61	1011.125	8.714	553.738	7807.784	0.036	12.323
6846.409	55793.04	13005.125	113.728	578.613	193892.977	0.459	11.832
6861.514	55853.74	7895.125	69.042	1051.055	91598.558	0.279	6.528

6874.887	55907.41	4173.125	36.493	1002.846	43462.515	0.147	6.855
6901.473	56013.97	14748.125	128.971	795.684	164637.421	0.521	8.674
6917.311	56077.36	6794.125	59.414	989.481	62982.669	0.240	6.991
6938.174	56160.74	5296.125	46.314	790.379	61049.686	0.187	8.778
6961.460	56253.65	28321.125	250.890	763.153	340781.862	1.000	9.122
6977.522	56317.65	16669.625	147.672	811.612	200973.017	0.589	8.597
7003.642	56421.58	1317.875	11.675	1265.635	8973.889	0.047	5.534
7011.539	56452.96	1637.875	14.510	782.048	23399.506	0.058	8.966
7060.375	56646.63	1325.375	11.741	445.900	17335.822	0.047	15.834
7070.183	56685.44	1488.625	13.187	712.303	17693.517	0.053	9.926
7073.719	56699.43	1472.375	13.192	710.619	17693.517	0.052	9.954
7083.411	56737.74	1229.875	11.020	2478.748	8500.103	0.043	2.858
7090.850	56767.14	923.875	8.278	1081.801	6101.906	0.033	6.555
7106.505	56828.95	1049.875	9.407	642.079	13328.465	0.037	11.068

Table S2. Supplementary Data from MALDI-TOF Analysis of PAMAM G3.0 Using THAP matrix.

m/z	time	Intens.	SN	Res.	Area	Rel. Intens.	FWHM
4989.908	47825.33	1231.000	4.061	697.115	9743.227	0.025	7.158
5005.425	47898.49	952.000	3.141	648.534	7520.902	0.020	7.718
5137.771	48517.88	1312.000	4.447	1310.965	8646.440	0.027	3.919
5141.988	48537.49	1221.000	4.139	1126.541	8095.952	0.025	4.564
5162.130	48631.01	945.000	3.203	893.218	8720.232	0.020	5.779
5179.596	48711.97	1042.000	3.532	450.593	13120.528	0.022	11.495
5198.596	48799.87	2477.000	8.554	686.522	24666.270	0.051	7.572
5203.777	48823.82	2262.000	7.812	951.130	21698.306	0.047	5.471
5217.143	48885.53	1947.000	6.724	1557.851	10581.504	0.040	3.349
5225.449	48923.84	1890.000	6.527	883.424	16339.838	0.039	5.915
5237.790	48980.71	2140.000	7.390	758.796	27901.063	0.044	6.903
5253.201	49051.63	2149.000	7.421	597.406	27867.471	0.045	8.793
5278.086	49165.93	1561.000	5.391	620.782	16012.378	0.032	8.502
5294.798	49242.53	1350.000	4.754	680.086	13427.632	0.028	7.785
5310.961	49316.51	1547.000	5.447	375.312	23228.682	0.032	14.151
5333.636	49420.10	1240.000	4.366	792.380	14091.212	0.026	6.731
5347.964	49485.44	1092.000	3.845	804.946	8640.782	0.023	6.644
5366.425	49569.51	2925.000	10.300	450.784	44420.333	0.061	11.905
5391.164	49681.93	2321.000	8.333	547.620	31687.609	0.048	9.845
5405.502	49746.97	2212.000	7.942	712.027	24207.675	0.046	7.592
5422.510	49824.01	4111.000	14.759	491.098	56969.038	0.085	11.042
5446.150	49930.89	3556.000	12.767	718.906	38602.284	0.074	7.576
5462.773	50005.91	3426.000	12.300	852.812	37150.998	0.071	6.406
5482.527	50094.91	10985.000	39.438	479.653	149433.778	0.227	11.430
5506.263	50201.63	10146.000	37.170	623.595	116685.013	0.210	8.830
5522.287	50273.55	8040.000	29.455	678.365	87835.821	0.166	8.141
5544.933	50375.02	1066.000	3.905	626.844	9598.977	0.022	8.846
5819.720	51590.04	807.500	3.155	563.863	7730.324	0.017	10.321
5879.828	51851.96	2186.500	8.542	464.783	29096.822	0.045	12.651
5903.216	51953.52	2079.000	8.122	660.679	20777.994	0.043	8.935
5919.920	52025.92	1588.500	6.334	655.736	16690.412	0.033	9.028
5939.555	52110.91	1317.250	5.252	625.449	9138.062	0.027	9.496
5962.785	52211.27	962.500	3.838	640.054	3981.230	0.020	9.316
5978.723	52280.01	1075.500	4.288	1011.643	6162.064	0.022	5.910
5999.855	52371.02	1612.250	6.428	517.133	17764.356	0.033	11.602
6023.513	52472.71	1740.500	6.940	682.047	16827.005	0.036	8.832
6038.387	52536.54	1419.500	5.759	749.449	13140.716	0.029	8.057
6107.449	52831.90	1060.500	4.302	404.746	13868.591	0.022	15.090
6133.009	52940.79	787.000	3.193	865.426	7986.802	0.016	7.087
6147.058	53000.55	833.250	3.436	654.754	7688.675	0.017	9.388
6167.422	53087.04	1745.500	7.199	515.132	16146.925	0.036	11.973
6190.406	53184.49	1080.500	4.456	599.143	4776.332	0.022	10.332

6206.728	53253.58	1102.000	4.545	883.281	6019.005	0.023	7.027
6221.931	53317.86	1403.125	5.787	534.923	11995.814	0.029	11.631
6281.171	53567.56	3515.000	14.709	516.466	42125.568	0.073	12.162
6304.068	53663.76	3017.250	12.626	661.198	28709.100	0.062	9.534
6319.508	53728.53	2091.000	8.750	1028.967	16447.489	0.043	6.142
6336.951	53801.61	5412.000	22.647	562.565	65038.675	0.112	11.264
6359.487	53895.87	5425.000	23.105	786.540	51915.365	0.112	8.085
6375.954	53964.65	5086.000	21.661	896.777	54127.175	0.105	7.110
6396.392	54049.89	14269.000	60.771	540.891	191283.960	0.295	11.826
6419.733	54147.06	14389.000	61.282	754.949	157751.816	0.298	8.504
6435.494	54212.58	11293.000	48.096	761.159	123460.501	0.234	8.455
6455.980	54297.62	3002.000	12.785	672.223	30217.180	0.062	9.604
6478.803	54392.20	2297.000	9.911	691.727	19614.195	0.048	9.366
6494.474	54457.04	1955.000	8.436	765.443	19086.711	0.040	8.485
6550.415	54687.89	1037.500	4.477	774.651	4818.356	0.021	8.456
6569.382	54765.93	2539.500	10.958	521.444	33892.278	0.053	12.598
6591.449	54856.59	2508.750	10.836	667.328	32774.252	0.052	9.877
6607.201	54921.22	2615.750	11.298	791.224	29474.904	0.054	8.351
6623.674	54988.71	4085.750	17.648	620.011	54225.963	0.085	10.683
6648.109	55088.68	3304.750	14.274	718.439	35365.691	0.068	9.254
6663.945	55153.37	3581.750	15.471	1013.917	36848.186	0.074	6.572
6684.063	55235.44	7798.750	33.686	546.355	123990.047	0.162	12.234
6707.455	55330.71	8384.750	36.217	805.330	110231.211	0.174	8.329
6724.141	55398.56	7067.750	30.528	938.281	82497.886	0.146	7.166
6737.010	55450.84	8197.750	35.409	596.253	125631.347	0.170	11.299
6761.521	55550.27	8735.750	37.733	773.177	112093.028	0.181	8.745
6778.017	55617.09	8485.750	36.653	1056.674	97981.365	0.176	6.414
6797.296	55695.07	19447.750	84.002	546.757	333717.663	0.403	12.432
6821.048	55790.99	21295.750	91.984	746.064	314963.525	0.441	9.143
6837.066	55855.59	19900.750	85.959	1024.233	243242.437	0.412	6.675
6852.769	55918.84	30526.750	131.856	645.717	468388.126	0.632	10.613
6876.618	56014.77	30835.750	133.191	795.080	397275.501	0.639	8.649
6892.484	56078.50	28535.750	123.256	892.422	349036.285	0.591	7.723
6913.485	56162.73	46578.750	201.191	580.396	735312.988	0.965	11.912
6936.708	56255.73	48288.750	208.577	757.715	625265.003	1.000	9.155
6952.436	56318.63	39682.750	171.404	715.074	569862.387	0.822	9.723
6989.018	56464.64	2133.750	9.216	940.825	17803.117	0.044	7.429
7006.695	56535.06	2190.750	9.463	687.646	22797.164	0.045	10.189
7031.884	56635.26	1246.750	5.385	765.512	7114.267	0.026	9.186
7049.364	56704.68	961.000	4.151	701.442	7987.665	0.020	10.050
7067.697	56777.40	2395.250	10.346	658.285	29350.952	0.050	10.737
7090.649	56868.31	1938.500	8.373	512.645	30869.345	0.040	13.831
7149.895	57102.29	875.250	3.781	443.361	10110.463	0.018	16.127
7179.730	57219.75	1015.000	4.401	804.034	12297.805	0.021	8.930

7198.769	57294.58	716.750	3.108	718.067	7053.525	0.015	10.025
7217.250	57367.12	811.000	3.516	1060.180	5955.570	0.017	6.808
13322.549	77661.55	434.000	5.887	1507.758	5371.190	0.009	8.836
13669.486	78655.18	435.000	6.013	872.845	7663.532	0.009	15.661
13679.598	78683.95	420.000	5.805	2105.136	3272.235	0.009	6.498
13697.962	78736.17	433.000	6.042	1137.384	4991.075	0.009	12.043
13704.371	78754.39	436.000	6.084	1540.714	3965.839	0.009	8.895
13723.516	78808.77	494.000	6.893	1141.143	7155.757	0.010	12.026
13755.656	78899.99	436.000	6.084	1931.924	3072.626	0.009	7.120
13763.756	78922.96	456.000	6.363	1557.285	4029.563	0.009	8.838
13782.695	78976.65	503.000	7.019	1589.410	6056.317	0.010	8.672
13796.297	79015.19	448.000	6.251	3290.606	4171.581	0.009	4.193
13818.333	79077.58	406.000	5.665	1511.175	5216.537	0.008	9.144
13842.091	79144.78	424.000	5.917	1102.419	4104.128	0.009	12.556

Table S3. Supplementary Data from MALDI-TOF Analysis of PAMAM G3.0 Using SA.

m/z	time	Intens.	SN	Res.	Area	Rel. Intens.	FWHM
4988.596	47819.14	1559.500	4.365	856.717	12610.296	0.061	5.823
5002.735	47885.81	1484.500	4.155	650.152	14133.577	0.058	7.695
5178.718	48707.90	1055.250	3.018	551.946	10690.026	0.041	9.383
5194.114	48779.15	1112.250	3.245	542.531	12311.266	0.044	9.574
5224.305	48918.57	1877.250	5.477	544.642	24131.093	0.073	9.592
5238.872	48985.69	1670.250	4.873	620.989	19487.749	0.065	8.436
5275.359	49153.42	1232.250	3.595	586.976	14672.746	0.048	8.987
5294.242	49239.98	1345.250	4.012	617.961	10575.512	0.053	8.567
5335.178	49427.14	1496.250	4.462	454.470	18645.884	0.059	11.739
5347.749	49484.46	1211.250	3.612	819.370	9647.017	0.047	6.527
5391.979	49685.63	2160.250	6.548	612.288	27501.491	0.085	8.806
5406.457	49751.30	2388.250	7.240	620.958	27765.331	0.093	8.707
5418.584	49806.24	1432.250	4.342	1003.738	10090.863	0.056	5.398
5445.730	49929.00	3354.250	10.168	724.751	37927.508	0.131	7.514
5462.067	50002.72	3101.250	9.401	545.506	40121.739	0.121	10.013
5480.684	50086.61	1711.250	5.187	495.604	22394.796	0.067	11.059
5505.799	50199.55	7640.250	23.581	660.571	85877.482	0.299	8.335
5521.743	50271.11	6928.250	21.384	671.776	79288.315	0.271	8.220
5711.913	51116.84	1031.000	3.299	650.706	7216.051	0.040	8.778
5728.394	51189.47	1229.000	3.932	1223.153	8685.353	0.048	4.683
5905.034	51961.40	1740.000	5.674	521.727	19756.164	0.068	11.318
5917.556	52015.68	1773.000	5.880	509.323	23240.053	0.069	11.618
5962.246	52208.94	1076.500	3.570	732.208	7270.980	0.042	8.143
5977.679	52275.51	1162.500	3.856	660.819	7978.288	0.045	9.046
6023.087	52470.88	1397.500	4.635	706.503	15626.986	0.055	8.525
6037.341	52532.06	1596.000	5.374	570.278	17238.835	0.062	10.587
6135.226	52950.23	1066.500	3.646	583.198	11397.449	0.042	10.520
6189.548	53180.86	1163.000	3.976	729.726	8093.934	0.046	8.482
6205.852	53249.88	1114.000	3.808	700.355	8423.376	0.044	8.861
6247.234	53424.66	1180.500	4.097	520.274	15599.613	0.046	12.008
6304.159	53664.14	2480.000	8.607	649.809	24755.464	0.097	9.702
6319.886	53730.11	2374.500	8.241	451.315	32240.675	0.093	14.003
6359.425	53895.62	3703.000	13.028	646.839	38457.145	0.145	9.832
6375.781	53963.93	3642.000	12.813	783.704	39036.879	0.142	8.135
6393.675	54038.56	1453.000	5.112	510.168	15723.214	0.057	12.532
6397.504	54054.52	1408.000	4.953	503.343	15723.214	0.055	12.710
6419.331	54145.39	9071.000	31.913	756.926	100887.761	0.355	8.481
6435.234	54211.50	8068.000	28.384	751.029	89217.478	0.316	8.569
6478.578	54391.26	1962.000	7.011	757.593	17895.134	0.077	8.552
6493.833	54454.39	1395.000	4.985	672.705	11796.977	0.055	9.653
6532.411	54613.70	1077.250	3.849	605.874	8411.264	0.042	10.782
6592.238	54859.83	2730.375	9.910	676.295	38140.283	0.107	9.748

6608.768	54927.64	2616.375	9.496	713.667	37457.029	0.102	9.260
6622.954	54985.76	2044.375	7.420	640.820	29586.219	0.080	10.335
6645.653	55078.64	3541.375	12.853	673.759	48809.658	0.139	9.864
6663.067	55149.78	2859.375	10.378	986.869	33441.511	0.112	6.752
6678.306	55211.96	1628.375	5.910	1656.468	10829.679	0.064	4.032
6688.679	55254.25	1572.375	5.707	1773.437	6919.179	0.062	3.772
6707.071	55329.14	5521.375	20.330	669.114	76577.641	0.216	10.024
6722.403	55391.50	4952.375	18.235	612.795	81144.644	0.194	10.970
6761.819	55551.48	5091.375	18.746	705.994	65978.781	0.199	9.578
6776.451	55610.74	5211.375	19.188	728.873	65504.641	0.204	9.297
6790.838	55668.96	3294.375	12.130	2303.730	31207.120	0.129	2.948
6804.824	55725.49	3287.375	12.104	1081.269	24900.661	0.129	6.293
6820.809	55790.03	12266.375	45.778	701.566	179250.904	0.480	9.722
6836.488	55853.26	11587.375	43.244	607.429	249383.919	0.453	11.255
6876.392	56013.86	16477.375	61.493	822.038	214881.043	0.645	8.365
6892.317	56077.83	15484.375	57.787	835.957	207609.037	0.606	8.245
6911.240	56153.73	6903.375	25.763	765.788	101635.852	0.270	9.025
6936.509	56254.94	25558.375	96.671	794.944	338206.980	1.000	8.726
6952.056	56317.11	22446.375	84.901	747.692	318615.110	0.878	9.298
6980.431	56430.41	1925.375	7.282	1668.432	13561.354	0.075	4.184
6986.940	56456.36	1626.375	6.152	887.230	14137.556	0.064	7.875
7030.763	56630.81	1609.375	6.087	626.068	14420.514	0.063	11.230
7046.040	56691.49	2032.375	7.687	480.335	34142.089	0.080	14.669
7083.011	56838.07	1198.375	4.586	692.732	10424.652	0.047	10.225
7099.419	56903.01	1655.375	6.335	978.012	11907.284	0.065	7.259
7142.493	57073.11	2743.875	10.500	807.633	27176.054	0.107	8.844
7158.968	57138.04	3900.125	14.925	944.605	49400.468	0.153	7.579
7173.636	57195.78	1853.125	7.167	800.940	21399.526	0.073	8.957
7190.348	57261.49	1008.375	3.900	735.548	9133.274	0.039	9.775

Table S4. Supplementary Data from MALDI-TOF Analysis of PAMAM G3.0 Using a combination of HCCA-SA

m/z	time	Intens.	SN	Res.	Area	Rel. Intens.	FWHM
4988.336	47817.91	772.000	5.044	748.732	6314.642	0.052	6.662
5004.166	47892.55	831.000	5.430	811.509	6298.777	0.056	6.166
5176.513	48697.68	600.500	4.028	809.825	4077.435	0.041	6.392
5219.848	48898.01	772.000	5.308	751.265	7107.299	0.052	6.948
5221.003	48903.34	740.000	5.088	551.034	7107.299	0.050	9.475
5234.590	48965.97	741.000	5.095	353.763	11882.487	0.050	14.797
5249.440	49034.33	497.000	3.417	1514.154	2004.537	0.034	3.467
5276.055	49156.61	681.000	4.683	851.542	6897.901	0.046	6.196
5292.673	49232.80	575.000	4.044	669.086	5917.695	0.039	7.910
5329.036	49399.10	487.000	3.425	1493.970	3124.611	0.033	3.567
5336.647	49433.84	569.000	4.002	786.001	4877.013	0.038	6.790
5350.078	49495.07	647.000	4.551	1708.981	3748.455	0.044	3.131
5354.056	49513.20	463.000	3.257	1513.126	2033.600	0.031	3.538
5362.731	49552.70	524.000	3.686	1060.217	3191.215	0.035	5.058
5388.520	49669.93	984.000	7.059	524.827	11670.451	0.066	10.267
5391.955	49685.52	984.000	7.059	534.147	11670.451	0.066	10.095
5405.867	49748.62	1128.000	8.092	686.730	12011.447	0.076	7.872
5419.196	49809.01	699.000	5.014	925.737	5950.353	0.047	5.854
5444.886	49925.18	1671.000	11.987	756.542	15844.764	0.113	7.197
5460.742	49996.75	1707.000	12.245	739.413	17992.968	0.115	7.385
5479.341	50080.56	1001.000	7.181	527.303	13275.981	0.068	10.391
5504.744	50194.81	3704.000	27.112	685.606	38848.102	0.250	8.029
5520.580	50265.89	3285.000	24.045	612.986	37703.215	0.222	9.006
5757.168	51316.02	436.500	3.321	679.359	3391.475	0.029	8.474
5844.271	51697.18	399.500	3.102	580.708	3047.255	0.027	10.064
5902.236	51949.27	830.500	6.448	617.059	8516.500	0.056	9.565
5919.134	52022.52	846.500	6.705	636.481	9130.610	0.057	9.300
5961.255	52204.66	782.750	6.200	981.953	4234.402	0.053	6.071
5977.702	52275.61	635.000	5.030	879.943	3952.356	0.043	6.793
6022.284	52467.43	752.750	5.962	642.417	6988.779	0.051	9.374
6033.974	52517.61	668.500	5.403	397.810	9814.934	0.045	15.168
6037.645	52533.36	693.500	5.605	400.392	9814.934	0.047	15.079
6133.169	52941.47	486.500	3.932	583.432	4085.422	0.033	10.512
6147.946	53004.32	644.000	5.300	883.998	5961.293	0.043	6.955
6160.703	53058.52	404.500	3.329	659.248	2534.207	0.027	9.345
6188.589	53176.79	601.000	4.946	779.542	3960.037	0.041	7.939
6204.925	53245.95	691.500	5.691	858.823	3889.854	0.047	7.225
6242.935	53406.53	661.500	5.444	909.103	4181.170	0.045	6.867
6260.028	53478.58	649.500	5.434	754.480	5069.456	0.044	8.297
6303.641	53661.97	1343.000	11.236	594.088	12331.674	0.091	10.611
6318.454	53724.11	1433.000	11.989	839.293	13909.243	0.097	7.528

6333.183	53785.83	392.500	3.284	661.772	1227.848	0.026	9.570
6359.158	53894.50	2166.500	18.450	772.686	17674.347	0.146	8.230
6374.617	53959.07	2181.500	18.578	728.437	22154.623	0.147	8.751
6392.992	54035.71	1009.500	8.597	761.418	9289.584	0.068	8.396
6418.287	54141.05	4930.500	41.989	775.662	48675.360	0.333	8.275
6434.161	54207.04	4554.500	38.787	800.274	47844.870	0.307	8.040
6477.063	54384.99	1128.500	9.780	674.442	11048.190	0.076	9.604
6493.972	54454.97	790.500	6.851	753.531	6514.767	0.053	8.618
6532.367	54613.52	585.500	5.074	808.818	4031.501	0.040	8.076
6545.319	54666.90	582.500	5.048	869.459	4862.445	0.039	7.528
6559.720	54726.19	641.500	5.560	747.682	5893.525	0.043	8.773
6572.780	54779.90	413.500	3.584	922.472	2356.760	0.028	7.125
6593.058	54863.20	1447.500	12.727	662.407	17635.449	0.098	9.953
6604.705	54910.98	1667.500	14.662	776.472	22064.983	0.113	8.506
6619.555	54971.84	1070.500	9.413	2660.104	5716.251	0.072	2.488
6625.450	54995.98	992.500	8.727	2429.668	4772.310	0.067	2.727
6633.636	55029.49	993.500	8.735	1081.253	5796.853	0.067	6.135
6647.095	55084.53	1702.500	14.969	874.786	19724.646	0.115	7.599
6661.237	55142.31	1887.500	16.596	864.637	21398.618	0.127	7.704
6673.675	55193.08	1554.500	13.668	1231.209	15699.222	0.105	5.420
6683.015	55231.16	1125.500	9.896	3617.585	6229.893	0.076	1.847
6691.267	55264.80	973.500	8.560	1686.671	3640.857	0.066	3.967
6706.211	55325.64	3244.500	28.781	769.536	41076.854	0.219	8.715
6721.693	55388.61	2732.500	24.239	595.403	47854.732	0.184	11.289
6760.725	55547.04	3286.500	29.153	850.820	38604.618	0.222	7.946
6776.127	55609.43	3176.500	28.177	904.670	38060.032	0.214	7.490
6789.372	55663.03	1749.500	15.519	2543.138	8965.013	0.118	2.670
6794.576	55684.07	1766.500	15.670	1007.000	19446.882	0.119	6.747
6820.096	55787.15	6576.500	58.857	627.881	96746.878	0.444	10.862
6835.748	55850.28	6559.500	58.705	740.609	94984.081	0.443	9.230
6848.377	55901.16	3578.500	32.026	1228.441	36695.195	0.242	5.575
6875.010	56008.31	9652.500	86.386	816.780	121059.181	0.652	8.417
6891.208	56073.37	9231.500	82.618	883.738	110051.702	0.623	7.798
6910.476	56150.67	3967.500	35.508	688.917	61349.827	0.268	10.031
6935.260	56249.94	14811.500	133.124	786.003	185669.500	1.000	8.823
6951.091	56313.25	13655.500	122.734	779.602	186892.067	0.922	8.916
6984.225	56445.54	1174.500	10.556	1773.218	6206.820	0.079	3.939
6998.657	56503.06	872.500	7.842	1406.335	6618.598	0.059	4.977
7010.743	56551.18	592.500	5.325	2696.555	2144.330	0.040	2.600
7017.976	56579.96	603.500	5.424	1335.977	3441.499	0.041	5.253
7030.180	56628.49	954.500	8.579	776.557	11515.227	0.064	9.053
7048.158	56699.90	1004.500	9.064	958.899	8174.905	0.068	7.350
7056.961	56734.83	1063.500	9.596	1081.159	10780.181	0.072	6.527
7076.012	56810.35	749.500	6.763	1771.641	5004.467	0.051	3.994

7083.736	56840.95	750.500	6.772	707.410	8501.284	0.051	10.014
7113.811	56959.90	1086.500	9.804	753.580	13259.154	0.073	9.440
7129.273	57020.96	1092.500	9.858	1105.913	9307.575	0.074	6.447
7142.618	57073.60	562.500	5.076	1744.060	2795.849	0.038	4.095
7150.519	57104.75	674.500	6.086	803.058	7435.826	0.046	8.904
7174.200	57198.00	1945.500	17.789	809.498	22161.361	0.131	8.863
7190.158	57260.75	1879.500	17.186	862.102	21028.139	0.127	8.340
7217.251	57367.12	767.750	7.020	632.616	11523.246	0.052	11.409
7230.567	57419.33	654.250	5.982	915.143	6056.175	0.044	7.901
7286.239	57637.09	427.500	3.963	947.403	3537.133	0.029	7.691
13733.460	78837.01	137.500	3.357	576.414	2201.464	0.009	23.826

Table S5. Supplementary Data from MALDI-TOF Analysis of PAMAM G3.0 Using a combination of THAP-HCCA.

m/z	time	Intens.	SN	Res.	Area	Rel. Intens.	FWHM
4988.775	47819.98	2610.500	3.575	702.941	18993.822	0.027	7.097
5004.752	47895.31	9773.500	13.386	592.013	98926.248	0.103	8.454
5064.297	48175.01	4258.500	5.833	713.378	35229.969	0.045	7.099
5125.151	48459.17	2767.000	3.923	456.029	30099.804	0.029	11.239
5178.801	48708.29	5735.000	8.131	592.688	60859.953	0.060	8.738
5191.538	48767.24	3035.000	4.441	754.770	30712.141	0.032	6.878
5203.655	48823.26	2763.000	4.043	702.792	27295.619	0.029	7.404
5223.001	48912.56	4210.000	6.160	752.918	40869.571	0.044	6.937
5239.085	48986.67	10712.000	15.673	445.707	196863.632	0.113	11.755
5276.725	49159.68	3653.000	5.345	920.096	34077.972	0.038	5.735
5293.536	49236.75	8610.000	12.952	379.023	147970.530	0.091	13.966
5321.649	49365.36	2778.000	4.179	853.836	27929.361	0.029	6.233
5351.362	49500.93	7373.000	11.091	809.837	77196.143	0.078	6.608
5359.326	49537.20	8384.000	12.612	636.152	118144.044	0.088	8.425
5406.072	49749.56	14133.000	21.808	562.054	195221.662	0.149	9.618
5419.289	49809.43	6835.000	10.547	780.078	70782.859	0.072	6.947
5446.334	49931.72	7934.000	12.243	774.983	92150.424	0.084	7.028
5461.866	50001.82	19467.000	30.039	693.252	215384.387	0.205	7.879
5482.447	50094.54	6498.000	10.027	601.297	87419.328	0.068	9.118
5506.119	50200.99	15943.000	25.285	743.699	159454.978	0.168	7.404
5521.861	50271.64	41636.000	66.032	653.820	444755.341	0.439	8.446
5581.982	50540.56	2528.000	4.009	416.043	31785.174	0.027	13.417
5635.732	50779.76	2182.750	3.548	687.615	17111.472	0.023	8.196
5693.447	51035.34	2058.500	3.346	626.022	16745.479	0.022	9.095
5719.138	51148.69	3070.500	5.115	717.135	28393.731	0.032	7.975
5759.001	51324.07	4383.500	7.303	349.302	60745.008	0.046	16.487
5804.118	51521.83	3791.375	6.316	507.339	51595.213	0.040	11.440
5816.937	51577.87	3298.750	5.621	692.716	30847.698	0.035	8.397
5859.597	51763.96	4765.000	8.119	631.749	47053.876	0.050	9.275
5903.656	51955.43	3425.000	5.836	873.696	27264.523	0.036	6.757
5919.178	52022.71	10888.000	18.971	604.537	128113.249	0.115	9.791
5962.669	52210.77	2169.000	3.779	805.693	13869.389	0.023	7.401
5978.660	52279.74	8616.000	15.013	723.157	77046.843	0.091	8.267
6022.286	52467.44	3393.250	5.912	1078.494	25050.336	0.036	5.584
6038.258	52535.99	10056.250	17.902	608.531	124802.800	0.106	9.923
6091.921	52765.64	5766.500	10.266	751.100	50980.406	0.061	8.111
6103.676	52815.81	2302.500	4.099	974.540	15807.805	0.024	6.263
6150.460	53015.01	6596.500	12.006	348.746	103661.909	0.069	17.636
6190.276	53183.94	2502.000	4.554	1008.199	12654.472	0.026	6.140
6206.665	53253.31	7574.000	13.786	581.335	86670.952	0.080	10.677
6261.829	53486.16	7723.500	14.328	686.831	115195.769	0.081	9.117
6273.221	53534.12	4402.500	8.167	631.685	28385.588	0.046	9.931

6305.048	53667.88	4498.500	8.345	797.772	27034.125	0.047	7.903
6320.108	53731.04	17636.500	32.718	658.569	202599.067	0.186	9.597
6333.717	53788.07	3518.500	6.527	926.744	20101.532	0.037	6.834
6359.909	53897.64	8421.000	15.928	958.850	65943.519	0.089	6.633
6375.651	53963.38	26615.000	50.341	698.700	296735.837	0.280	9.125
6394.914	54043.72	3969.000	7.507	805.990	44343.264	0.042	7.934
6419.602	54146.51	20293.000	38.383	889.677	188286.940	0.214	7.216
6435.199	54211.35	50151.000	94.858	694.748	551163.943	0.528	9.263
6479.013	54393.07	4384.000	8.455	846.061	33948.925	0.046	7.658
6495.334	54460.60	13503.000	26.040	732.715	141123.598	0.142	8.865
6532.848	54615.50	2228.000	4.297	937.344	10582.166	0.023	6.970
6549.272	54683.18	6546.000	12.624	845.975	53909.033	0.069	7.742
6560.184	54728.10	1986.000	3.830	1727.545	10926.120	0.021	3.797
6575.450	54790.88	2722.000	5.249	956.940	21411.348	0.029	6.871
6592.905	54862.57	5069.375	9.885	963.684	41386.081	0.053	6.841
6608.918	54928.25	16629.375	32.427	624.308	259233.137	0.175	10.586
6633.436	55028.67	7202.375	14.044	772.513	78937.760	0.076	8.587
6647.997	55088.22	5920.375	11.544	1045.694	47820.762	0.062	6.357
6663.817	55152.85	17208.375	33.556	744.762	268177.720	0.181	8.948
6691.254	55264.74	3844.375	7.496	1728.352	28396.828	0.040	3.871
6708.273	55334.04	14696.375	28.764	966.890	156299.411	0.155	6.938
6723.496	55395.94	31189.375	61.045	672.686	471073.170	0.329	9.995
6762.184	55552.96	12301.375	24.077	945.486	129290.917	0.130	7.152
6777.619	55615.47	31630.375	61.908	781.715	458174.695	0.333	8.670
6802.094	55714.46	7799.375	15.265	1858.752	52264.633	0.082	3.659
6822.045	55795.02	29417.375	58.008	865.659	334228.854	0.310	7.881
6836.673	55854.01	58902.375	116.150	569.138	1174240.645	0.620	12.012
6876.922	56015.99	37499.375	73.945	890.895	468133.802	0.395	7.719
6914.758	56167.84	20304.375	40.038	832.147	309546.216	0.214	8.310
6936.853	56256.31	55704.375	110.124	902.377	677636.353	0.587	7.687
6951.961	56316.73	94930.375	187.671	616.301	1546405.676	1.000	11.280
6989.400	56466.17	7509.375	14.846	596.445	136546.189	0.079	11.718
7035.474	56649.52	5646.375	11.163	654.981	73875.169	0.059	10.741
7049.872	56706.70	5184.375	10.253	1021.058	50339.701	0.055	6.904
7067.045	56774.82	3279.375	6.486	993.694	34506.908	0.035	7.112
7077.205	56815.08	2834.375	5.606	1879.002	21586.758	0.030	3.766
7090.363	56867.18	5924.375	11.717	618.784	50094.335	0.062	11.459
7130.207	57024.64	4778.875	9.451	657.541	24288.493	0.050	10.844
7151.237	57107.58	12050.875	23.834	625.089	133775.215	0.127	11.440
7190.986	57264.00	13168.375	26.409	352.487	245282.341	0.139	20.401
7221.527	57383.89	2558.875	5.132	933.575	27279.727	0.027	7.735
7233.411	57430.48	3105.875	6.229	566.352	47892.470	0.033	12.772
7296.899	57678.69	1555.938	3.163	445.828	29060.098	0.016	16.367
13225.790	77382.11	384.000	3.181	709.962	6154.378	0.004	18.629

13743.774	78866.28	476.000	4.061	498.638	14702.336	0.005	27.563
13790.872	78999.82	353.000	3.011	2220.164	2591.585	0.004	6.212
13798.137	79020.40	408.000	3.481	1317.580	4732.690	0.004	10.472

Table S6. Supplementary Data from MALDI-TOF Analysis of PAMAM G3.0 Using a combination of THAP-SA

m/z	time	Intens.	SN	Res.	Area	Rel. Intens.	FWHM
4992.042	47835.39	3414.500	7.474	569.898	35616.925	0.049	8.760
5008.418	47912.58	2798.500	6.126	648.467	26466.258	0.040	7.723
5051.589	48115.46	1733.500	3.795	679.774	12799.032	0.025	7.431
5124.465	48455.97	1502.000	3.390	473.439	18398.376	0.022	10.824
5165.878	48648.39	2758.500	6.226	663.879	26388.535	0.040	7.781
5180.510	48716.20	2526.500	5.703	716.303	30832.359	0.036	7.232
5195.748	48786.71	1418.500	3.296	989.796	8900.969	0.020	5.249
5226.161	48927.13	5106.500	11.867	651.210	59180.558	0.073	8.025
5240.828	48994.70	4825.500	11.214	593.652	65231.029	0.069	8.828
5251.050	49041.74	2834.500	6.587	797.748	23460.623	0.041	6.582
5263.025	49096.78	1697.500	3.945	2862.615	7005.223	0.024	1.839
5280.495	49176.98	4161.500	9.671	795.279	41561.715	0.060	6.640
5295.890	49247.53	2759.500	6.577	682.529	30869.954	0.040	7.759
5308.049	49303.19	1332.500	3.176	1544.480	4258.698	0.019	3.437
5311.165	49317.44	1316.500	3.138	948.549	9195.784	0.019	5.599
5324.254	49377.26	1679.500	4.003	944.435	11455.983	0.024	5.638
5337.951	49439.79	3321.500	7.917	530.753	49391.129	0.048	10.057
5353.622	49511.22	2852.500	6.799	919.690	30800.557	0.041	5.821
5367.996	49576.66	1353.500	3.226	1184.599	7282.779	0.019	4.531
5394.483	49696.99	6281.500	15.289	652.515	75706.429	0.090	8.267
5409.387	49764.58	4733.500	11.522	701.414	51659.019	0.068	7.712
5420.971	49817.04	2094.500	5.098	1482.746	17860.593	0.030	3.656
5449.317	49945.19	8002.500	19.478	671.392	94439.317	0.115	8.116
5465.436	50017.91	5838.500	14.211	714.724	65368.496	0.084	7.647
5484.550	50104.01	2469.500	6.011	502.029	33979.891	0.036	10.925
5509.416	50215.79	21932.500	54.660	663.310	251834.464	0.316	8.306
5525.084	50286.09	15926.500	39.692	693.058	175392.488	0.229	7.972
5568.535	50480.54	1664.500	4.148	427.325	20915.394	0.024	13.031
5793.426	51475.03	1557.250	4.065	439.850	16594.983	0.022	13.171
5807.166	51535.16	1221.750	3.189	1003.104	8447.046	0.018	5.789
5849.287	51719.04	1664.000	4.436	581.812	18921.247	0.024	10.054
5862.652	51777.26	1436.750	3.830	606.243	15404.292	0.021	9.670
5906.678	51968.53	5335.000	14.222	648.151	65186.220	0.077	9.113
5922.482	52037.02	3970.750	10.806	694.492	46737.367	0.057	8.528
5965.648	52223.62	4212.000	11.463	748.232	36932.666	0.061	7.973
5981.855	52293.51	2761.250	7.514	842.355	22670.187	0.040	7.101
6025.514	52481.30	5383.500	14.967	629.148	67121.139	0.077	9.577
6041.649	52550.53	3976.250	11.054	637.577	43123.392	0.057	9.476
6079.512	52712.63	2403.750	6.683	690.622	15777.709	0.035	8.803
6095.664	52781.62	1807.625	5.025	849.054	13411.011	0.026	7.179
6139.121	52966.80	3129.125	8.861	689.173	35875.743	0.045	8.908

6151.762	53020.54	3418.625	9.680	1035.404	35255.466	0.049	5.941
6165.100	53077.19	1435.375	4.064	852.918	10189.053	0.021	7.228
6194.314	53201.04	4144.125	11.735	833.834	33597.620	0.060	7.429
6209.822	53266.67	2430.125	6.881	822.228	20425.037	0.035	7.552
6249.391	53433.75	3480.125	10.020	544.282	42528.723	0.050	11.482
6264.858	53498.92	2543.000	7.322	867.973	23117.632	0.037	7.218
6308.239	53681.27	8888.500	25.593	659.676	93585.031	0.128	9.563
6324.064	53747.63	6498.500	18.711	683.631	68053.673	0.094	9.251
6363.261	53911.64	12695.000	37.212	687.258	134006.524	0.183	9.259
6378.910	53976.99	9387.000	27.515	779.039	99382.852	0.135	8.188
6396.647	54050.95	2226.000	6.525	984.561	20602.168	0.032	6.497
6422.724	54159.50	29297.000	85.876	698.026	355313.803	0.422	9.201
6438.685	54225.83	21343.000	62.561	788.741	224625.561	0.307	8.163
6482.532	54407.63	7773.000	23.114	718.268	83533.745	0.112	9.025
6497.856	54471.03	4526.000	13.459	748.433	40672.723	0.065	8.682
6536.786	54631.74	3509.000	10.435	783.110	24908.372	0.051	8.347
6551.781	54693.51	2160.000	6.423	958.825	16656.223	0.031	6.833
6596.535	54877.47	8834.000	26.355	655.634	128028.724	0.127	10.061
6611.248	54937.80	7383.000	22.026	665.718	137736.070	0.106	9.931
6651.425	55102.23	9165.000	27.343	771.218	122850.936	0.132	8.625
6666.714	55164.67	6117.000	18.249	798.800	69619.189	0.088	8.346
6684.712	55238.08	3022.000	9.016	878.985	33434.395	0.043	7.605
6711.130	55345.66	19845.000	59.206	658.559	320605.284	0.286	10.191
6726.224	55407.03	13144.000	39.214	730.767	195658.505	0.189	9.204
6765.444	55566.17	17619.000	52.565	705.805	253705.686	0.254	9.585
6780.867	55628.62	14126.000	42.144	861.332	201425.845	0.203	7.873
6824.627	55805.44	38108.000	113.692	596.399	677898.714	0.548	11.443
6839.652	55866.01	30361.000	90.579	694.692	576336.337	0.437	9.846
6879.690	56027.12	48046.000	143.341	746.527	726356.842	0.691	9.216
6895.551	56090.81	36633.000	109.291	816.452	495767.489	0.527	8.446
6914.143	56165.37	15536.000	46.350	1103.543	141321.665	0.224	6.265
6939.304	56266.12	69482.000	207.293	723.068	1143205.860	1.000	9.597
6955.166	56329.54	53778.000	160.442	703.475	795700.222	0.774	9.887
6991.165	56473.20	3643.000	10.869	578.214	34911.539	0.052	12.091
7037.831	56658.89	2772.500	8.271	630.478	26575.910	0.040	11.163
7052.992	56719.08	3399.500	10.142	727.968	41234.737	0.049	9.689
7089.532	56863.89	2093.750	6.247	582.220	27142.052	0.030	12.177
7101.577	56911.54	1779.750	5.310	697.192	16341.603	0.026	10.186
7147.818	57094.10	1846.750	5.510	845.360	15198.272	0.027	8.455
7161.855	57149.41	3255.500	9.712	706.378	62996.591	0.047	10.139
7180.480	57222.70	2080.750	6.247	1119.983	21506.280	0.030	6.411
7196.282	57284.81	1013.500	3.043	940.839	11010.462	0.015	7.649
13226.149	77383.15	397.125	4.343	1309.677	6111.345	0.006	10.099
13269.305	77507.91	394.125	4.310	1885.502	4542.277	0.006	7.038

13328.979	77680.08	428.125	4.682	1100.058	7661.789	0.006	12.117
13344.209	77723.96	437.125	4.780	1330.027	5899.179	0.006	10.033
13502.609	78178.85	396.125	4.360	841.441	7094.898	0.006	16.047
13607.141	78477.57	437.125	4.843	1839.186	3937.516	0.006	7.398
13635.506	78558.43	423.125	4.688	2576.463	4671.561	0.006	5.292
13673.293	78666.01	526.125	5.830	957.594	10215.279	0.008	14.279
13682.427	78692.00	477.125	5.287	1101.527	4848.129	0.007	12.421
13694.770	78727.09	425.125	4.710	1280.031	4133.122	0.006	10.699
13729.906	78826.92	639.125	7.132	649.318	18498.079	0.009	21.145
13784.138	78980.74	569.125	6.351	1311.407	10132.945	0.008	10.511
13786.079	78986.24	587.125	6.551	1311.592	10132.945	0.008	10.511
13804.729	79039.07	482.125	5.380	1484.502	4442.859	0.007	9.299
13816.589	79072.64	390.125	4.353	2364.384	2054.808	0.006	5.844
13846.973	79158.59	509.125	5.681	814.879	11045.601	0.007	16.993
13862.457	79202.35	419.125	4.716	2388.326	4355.795	0.006	5.804

Table S7. Supplementary Data from MALDI-TOF Analysis of PAMAM G4.0 Using HCCA as Matrix

m/z	time	Intens.	SN	Res.	Area	Rel. Intens.	FWHM
5377.215	49529.78	1057.422	2.218	4987.612	625.213	0.789	1.078
5896.967	51833.42	1099.375	2.428	5322.294	1065.252	0.820	1.108
6813.142	55659.14	1340.938	3.118	4290.292	1750.827	1.000	1.588
8644.243	62599.18	407.000	1.130	6441.536	373.907	0.304	1.342
9969.180	67168.78	367.250	1.193	7748.257	629.584	0.274	1.287
10371.041	68493.58	415.250	1.478	10579.223	344.338	0.310	0.980
12315.384	74566.26	372.375	1.638	19544.999	174.427	0.278	0.630

Table S8. Supplementary Data from MALDI-TOF Analysis of PAMAM G4.0 Using THAP as Matrix

m/z	time	Intens.	SN	Res.	Area	Rel. Intens.	FWHM
5505.753	50109.49	348.000	6.233	5142.864	341.509	0.476	1.071
6423.131	54064.14	342.000	6.572	8331.591	240.147	0.468	0.771
6751.677	55410.86	11.711	2.325	7805.800	59.073	0.016	0.865
6821.182	55691.53	731.000	14.315	5031.200	548.715	1.000	1.356
7123.345	56895.36	11.509	2.793	8078.099	59.919	0.016	0.882
7338.269	57736.12	626.000	12.265	6253.196	502.126	0.856	1.174
8773.096	63058.36	96.000	2.126	7157.204	118.254	0.131	1.226
10825.643	69961.50	89.000	2.285	2724.026	268.075	0.122	3.974
10885.597	70152.77	99.000	2.565	10836.981	86.386	0.135	1.004
13516.541	78077.95	125.875	4.243	8043.903	186.520	0.172	1.680
13629.610	78400.26	170.875	5.800	6057.872	289.452	0.234	2.250
14145.654	79854.42	174.875	6.062	8285.800	184.597	0.239	1.707

Table S9. Supplementary Data from MALDI-TOF Analysis of PAMAM G4.0 Using a Combination of HCCA-THAP.

m/z	time	Intens.	SN	Res.	Area	Rel. Intens.	FWHM
5896.912	51833.18	1305.313	4.559	5322.392	1272.127	0.473	1.108
6817.472	55676.58	2761.625	10.088	1716.680	8171.068	1.000	3.971
8734.356	62920.67	366.844	1.578	6474.906	410.036	0.133	1.349
10369.388	68488.18	514.000	2.613	5289.192	790.976	0.186	1.960
12545.477	75251.90	387.000	2.580	7752.073	517.875	0.140	1.618
14108.099	79749.51	274.313	2.275	6162.257	479.682	0.099	2.289

Table S10. Supplementary Data from MALDI-TOF Analysis of PAMAM G5.0 Using HCCA as Matrix.

m/z	time	Intens.	SN	Res.	Area	Rel. Intens.	FWHM
10869.055	70694.04	195.000	1.919	2711.514	597.265	0.889	4.008
13655.984	79084.46	149.000	1.514	2025.446	635.007	0.680	6.742
14359.215	81061.84	182.000	1.867	3590.201	797.119	0.830	4.000
23940.959	104274.85	170.250	2.076	2678.750	942.864	0.777	8.937
25025.507	106578.85	219.250	2.715	4107.698	1062.608	1.000	6.092
26078.211	108767.68	190.250	2.399	5689.296	833.244	0.868	4.584
27832.757	112319.39	95.000	1.231	5921.624	283.561	0.433	4.700

27977.566	112607.43	126.500	1.645	3726.810	1026.878	0.577	7.507
28736.711	114105.35	124.000	1.656	4400.243	603.819	0.566	6.531

Table S11. Supplementary Data from MALDI-TOF Analysis of PAMAM G5.0 Using a Combination of HCCA-THAP

m/z	time	Intens.	SN	Res.	Area	Rel. Intens.	FWHM
10311.244	68889.29	206.500	1.191	3396.852	673.464	0.455	3.036
10581.473	69769.55	245.500	1.421	3250.733	917.381	0.541	3.255
10899.140	70790.04	238.500	1.382	2639.124	848.492	0.526	4.130
12970.290	77106.55	453.500	2.693	2477.097	1629.205	1.000	5.236
13736.923	79314.61	254.250	1.512	4533.109	450.781	0.561	3.030
14178.438	80558.25	281.250	1.679	2118.318	1852.457	0.620	6.693
14518.733	81503.57	368.750	2.211	1566.163	2124.182	0.813	9.270
14559.437	81615.90	286.500	1.718	2090.967	1603.452	0.632	6.963
24951.262	106422.74	316.750	2.292	2741.451	1925.458	0.698	9.101
25071.208	106674.82	388.750	2.817	2983.271	2472.579	0.857	8.404
25576.719	107730.60	354.750	2.587	3340.411	3217.237	0.782	7.657
27640.459	111935.72	247.250	1.854	5043.407	1259.610	0.545	5.481
27948.683	112550.04	242.750	1.828	3082.280	1523.011	0.535	9.068
28355.816	113356.30	231.250	1.746	5130.566	1417.975	0.510	5.527
